# Clinical characteristics of peripheral neuropathy and risk factors in rheumatoid arthritis patients: A retrospective study

**DOI:** 10.1097/MD.0000000000047156

**Published:** 2026-01-16

**Authors:** SiHui He, Sha-Sha Hu, Jingjing Xie, Jianyong Zhang, Hongling Geng, Ertao Jia

**Affiliations:** a Department of Rheumatism, Shenzhen Traditional Chinese Medicine Hospital, Shenzhen, Guangdong, China; b Department of Rheumatism, The Fourth Clinical Medicine College of Guangzhou, University of Chinese Medicine, Shenzhen Traditional Chinese Medicine Hospital, Shenzhen, Guangdong, China; c Department of Gynecology, Guangdong Provincial Hospital of Chinese Medicine, The Second Affiliated Hospital of Guangzhou University of Chinese Medicine, Guangzhou, China; d Department of Rheumatism, The Fifth Clinical College of Guangzhou University of Chinese Medicine, Guangdong Second Hospital of Traditional Chinese Medicine, Guangzhou, China.

**Keywords:** anti-cyclic citrullinated peptide antibodies, clinical characteristics, peripheral neuropathy, rheumatoid arthritis, risk factors

## Abstract

To examine the clinical features and possible risk factors of rheumatoid arthritis (RA) patients with peripheral neuropathy (PN). This study retrospectively analyzed data from 183 RA patients using electronic medical records from January 2018 to December 2022 at Shenzhen Traditional Chinese Medicine Hospital. Among them, 8 patients exhibited involvement of multiple mononeuropathy, and 24 patients showed involvement of a single neuropathy. The study compared characteristics between RA patients with and without PN and investigated potential risk factors for PN. Patients with PN were found to be older (*P* < .05) compared to those without PN, particularly in multiple mononeuropathy patients. The most common symptoms of PN were pain (53.13%) and numbness (46.88%), followed by fatigue (15.63%) and burning sensations (12.50%). Electromyography results indicated that 84.38% of patients with PN experienced both sensory and motor impairment, 3.13% had only motor impairment, and 12.50% had only sensory impairment. Carpal tunnel syndrome was observed in 34.38% of patients with PN. Binary logistic regression analysis revealed age (*P* = .044) and anti-cyclic citrullinated peptide (anti-CCP) antibody level (*P* = .049) as significant predictors of PN in RA patients. Age and anti-CCP antibody levels emerged as risk factors for PN. PN typically manifests as peripheral nerve symptoms like pain and numbness in RA patients, with a higher incidence of carpal tunnel syndrome.

## 1. Introduction

Rheumatoid arthritis (RA) is a chronic autoimmune disease affecting multiple systems, with a prevalence ranging from 0.5% to 1%.^[1]^ It is primarily characterized by progressive inflammatory arthritis and often presents with extra-articular manifestations. Peripheral neuropathy (PN) is a common extra-articular manifestation, occurring in approximately 10% to 40% of RA patients.^[2]^ PN can manifest as pain, numbness, tingling, muscle weakness, and may even lead to foot drop and muscle atrophy. In RA, the immune system may erroneously target peripheral nerves, and medications such as nonsteroidal anti-inflammatory drugs, disease-modifying antirheumatic drugs, and biological agents can also induce PN.^[3-6]^

The clinical presentation of PN in RA patients varies, ranging from involvement of a single neuropathy (SN) to multiple mononeuropathy (MN). However, the risk factors and clinical characteristics associated with PN in RA patients remain poorly understood. This study aimed to investigate the clinical features and potential risk factors of PN in RA patients.

## 2. Study population

This retrospective study included 426 RA patients, of whom 183 were identified through electronic medical records from January 2018 to December 2022 at Shenzhen Traditional Chinese Medicine Hospital. All RA patients met the 2010 ACR/EULAR classification criteria and were aged 18 years or older. PN was defined according to AAEM/EFNS-PNS standards: Clinical symptoms (pain, numbness, or motor deficits) and electromyography-confirmed nerve dysfunction (sensory amplitude reduction > 30%, conduction velocity slowing > 20%, or prolonged distal latency). SN: single nerve distribution; MN: ≥2 noncontiguous nerves. Patients with severe cardiovascular, cerebrovascular, hepatic, or renal impairment, as well as those with dementia, pregnancy, psychiatric disorders, and PN attributable to other disorders such as diabetes, cancer, hypothyroidism, or amyloidosis, were excluded. The patients were divided into 2 groups: those with PN (32 patients) and those without PN (151 patients), including 8 with MN involvement and 24 with SN involvement.

## 3. Data collection

This study collected patients’ demographics and comprehensive clinical records, including age, gender, disease duration, laboratory findings, previous treatments, and clinical manifestations. Specifically, laboratory tests encompassed rheumatoid factor (RF), anti-cyclic citrullinated peptide (anti-CCP), C-reactive protein (CRP), and erythrocyte sedimentation rate (ESR). Previous treatments referred to patients’ history of receiving methotrexate (MTX), leflunomide (LEF), tumor necrosis factor-alpha (TNF-α) inhibitors, interleukin-6 (IL-6) inhibitors, or Janus kinase (JAK) inhibitors. Serological biomarkers were quantified at RA diagnosis, with all treatments initiated prior to PN onset, confirming the temporal sequence. Clinical manifestations, including neurological impairment of motor and sensory nerves analyzed through electromyography, were also documented, considering factors like amplitude, conduction velocity, distal onset latency, and F wave.

## 4. Disease assessment

RA disease activity was assessed using the 28-joint disease activity score based on ESR (DAS28-ESR), DAS28 based on CRP (DAS28-CRP), simple disease activity index, and clinical disease activity index. These evaluations relied on parameters such as ESR, CRP levels, swollen and tender joint counts in 28 joints, and patients’ self-assessment of overall well-being.

## 5. Statistical analysis

Statistical analyses were conducted using IBM SPSS Statistics, Version 23 (IBM Corp., Armonk). Continuous variables in demographic and medical data were presented as mean ± standard deviation. Independent-sample *t*-tests were employed for between-group comparisons, with homogeneity of variance assessed by Levene’s test. Categorical data were expressed as ratios and analyzed using the chi-square (2-sided χ^2^) test. Furthermore, binary logistic regression analysis was utilized to examine the correlation between each risk factor and PN incidence. Statistical significance was determined at *P* ≤ .05.

## 6. Results

### 6.1. Demographic and clinical characteristics

Table 1 presents the patients’ demographic data, comprehensive clinical records including previous treatments, laboratory results, disease assessments, and statistical analyses. It is evident that the mean age of RA patients in the groups with and without PN was 58.28 ± 13.43 and 52.87 ± 12.13 years, respectively (*P* = .03). Patients with MN were older, with a mean age of 63.88 ± 12.09 years, compared to those with SN, with a mean age of 56.38 ± 13.52 years. The proportion of female patients was 84.4% and 86.8% in the groups with and without PN, respectively. However, the limited subgroup sample sizes constrain the statistical power to detect robust differences and warrant cautious interpretation of these comparisons. There was no significant difference in the gender ratio of RA patients or in disease duration (9.69 ± 9.83 vs 8.77 ± 8.57 years) between the 2 groups.

**Table 1:** Patients’ clinical characteristics.

Parameters	Patients with PN			RA patients without PN (N = 151)	*P* value*	*P* value**	*P* value***	*P* value
	MN (N = 8)	SN (N = 24)	Total (N = 32)					
Age (Mean ± SD)	63.88 ± 12.09	56.38 ± 13.52	58.25 ± 13.40	52.87 ± 12.13	.17	.20	**.01**	**.03**
Gender (female)	6	21	27	131	.78	1.00	.68	.94
Disease duration (Mean ± SD)	13.50 ± 7.73	8.42 ± 10.27	9.69 ± 9.83	8.77 ± 8.57	.21	.86	.13	.59
Previous treatment								
MTX	5	8	13	55	.30	.77	.27	.66
LEF	2	4	6	15	1.00	.53	.45	.26
TNFai	0	2	2	16	1.00	1.00	.71	.67
IL6i	0	0	1	3	.56	1.00	.49	1.00
JAKi	0	0	0	10	–	.41	1.00	.29
Laboratory results								
RF (IU/mL, Mean ± SD)	186.73 ± 207.52	159.12 ± 167.74	166.02 ± 75.35	173.04 ± 239.63	.71	.79	.87	.88
Anti-CCP (U/ml, Mean ± SD)	196.08 ± 157.81	57.69 ± 68.02	92.28 ± 112.98	165.92 ± 218.82	**.04**	**.00**	.70	**.01**
ESR (mm/h, Mean ± SD)	61.75 ± 25.54	47.92 ± 36.55	51.38 ± 34.28	50.34 ± 30.46	.33	.73	.30	.86
CRP (mg/L, Mean ± SD)	21.29 ± 20.95	11.17 ± 11.69	15.31 ± 20.73	20.82 ± 29.21	.23	.55	.63	.19
Disease assessment								
DAS28-ESR (Mean ± SD)	5.12 ± 1.921	4.75 ± 1.38	4.84 ± 1.51	4.55 ± 1.52	.55	.23	.30	.32
DAS28-CRP (Mean ± SD)	5.15 ± 1.71	4.89 ± 1.35	4.96 ± 1.42	4.50 ± 1.52	.67	.63	.25	.12
CDAI (Mean ± SD)	33.13 ± 27.10	28.50 ± 17.61	29.66 ± 20.00	24.25 ± 16.85	.66	.26	.39	.11
SDAI (Mean ± SD)	35.90 ± 26.98	29.62 ± 17.40	31.19 ± 19.91	26.50 ± 17.05	.55	.41	.36	.17

Values are given as the numbers or the mean ± standard deviation (Mean ± SD). Bold values indicate a statistically significant difference, defined as a *P*-value of <.05.

anti-CCP = anti-cyclic peptide containing citrulline, CDAI = clinical disease activity index, CRP = C-reactive protein, DAS28-CRP = the 28-joint disease activity score-C-reactive protein, DAS28-ESR = the 28-joint disease activity score-erythrocyte sedimentation rate, ESR = erythrocyte sedimentation rate, IL-6i = interleukin-6 inhibitor, JAKi = Janus kinase inhibitor, LEF = leflunomide, MN = multiple mononeuropathy, MTX = methotrexate, PN = peripheral neuropathy, RA = rheumatoid arthritis RF = rheumatoid factor, SDAI = the simple disease activity index, SN = single neuropathy, TNF-αi = tumor necrosis factor-α inhibitor.

* *P* value: comparison between MN group and SN group.

** *P* value: comparison between SN group and RA patients without PN group.

*** *P* value: comparison between patients with MN group and RA patients without PN group, *P* value: comparison between RA patients with and without PN group.

The level of anti-CCP was 92.28 ± 112.98 and 165.92 ± 218.82 U/mL (*P* < .05) in the groups with and without PN, respectively. Moreover, the level of anti-CCP was lower in the SN group compared to the MN group (57.69 ± 68.02 vs 196.08 ± 152.81 U/mL, *P* < .05), though the small subgroup sizes (n = 24 vs n = 8) limit definitive conclusions. No significant differences were observed in the levels of RF (166.02 ± 175.35 vs 173.04 ± 239.63 IU/mL), ESR (51.38 ± 34.28 vs 50.34 ± 30.46 mm/h), and CRP (15.31 ± 20.73 vs 20.82 ± 29.21 mg/L) between the groups with and without PN. Additionally, there were no significant differences in DAS28-ESR (4.84 ± 1.51 vs 4.55 ± 1.52 scores), DAS28-CRP (4.96 ± 1.42 vs 4.50 ± 1.52 scores), clinical disease activity index (29.66 ± 20.00 vs 24.25 ± 16.85 scores), and simple disease activity index (31.19 ± 19.91 vs 26.50 ± 17.05 scores) between the 2 groups. However, the limited sample size reduces power to detect subtle associations, and their potential role in PN pathogenesis cannot be excluded.

Regarding previous treatments, 13 (40.6%) and 55 (36.4%) patients were treated with MTX, 6 (18.8%) and 15 (9.9%) patients were treated with LEF, 2 (6.3%) and 16 (10.6%) patients were treated with TNF-α inhibitor (TNF-αi), 1 (3.1%) and 3 (2.0%) patients were treated with IL-6 inhibitor (IL-6i), and 0 and 10 (6.6%) patients were treated with JAK inhibitor (JAKi), respectively. Although treatments preceded PN onset, retrospective records limited precise tracking of exposure durations and cumulative doses, which may confound neurotoxicity evaluations.

### 6.2. Symptom presentation in RA patients with PN

The most commonly reported symptoms among PN patients were pain and numbness, with 53.13% experiencing pain and 46.88% experiencing numbness. Other reported symptoms included muscle weakness and a burning-like sensation. Electromyography results further confirmed sensory and motor impairments (see Table 2). Fatigue was reported by 15.63% of patients, while a burning-like sensation was observed in 12.50% of patients. Among the patients, 84.38% exhibited sensory-motor impairment, 3.13% had isolated motor impairment, and 12.50% had isolated sensory impairment. Carpal tunnel syndrome was the most prevalent condition, with a prevalence rate of 34.38%.

**Table 2:** The peripheral involvement features of RA patients with PN.

Parameters	RA patients with PN (N = 32)		
	MN (N = 8)	SN (N = 24)	Total (N = 32)
Symptom			
Pain	62.5%	50%	53.1%
Numbness	62.5%	41.7%	46.9%
Burning-like sensation	25%	12.5%	15.6%
Muscle weakness	37.5%	4.2%	12.5%
Involvement			
Pure sensory	0	16.7%	12.5%
Pure motor	0	4.2%	3.1%
Sensory-motor	100%	79.2%	84.4%
Carpal tunnel syndrome	0	45.8%	34.4%

Values are shown as a percentage (%).

MN = multiple mononeuropathy, PN = peripheral neuropathy, RA = rheumatoid arthritis, SN = single neuropathy.

### 6.3. The binary logistic regression analysis for predictors of risk factors with the occurrence of PN

Subsequently, the results of binary logistic regression analysis for predictors of risk factors of PN in RA patients are presented in Table 3. Notably, the analysis revealed that patient age (*P* = .044) and anti-CCP antibody levels (*P* = .049) were significant predictors of PN. However, gender, disease duration, treatment drugs, RF, ESR, CRP, and DAS28-ESR showed no significant associations.

**Table 3:** Binary logistic regression analysis for predictors of PN in RA patients.

Parameters	*B*	95% CI for *B*	*P* value
Age	0.039	1.001, 1.080	.044
Gender (female)	−0.285	0.238, 2.378	.628
Disease duration	−0.001	0.954, 1.046	.962
MTX	0.397	0.641, 3.450	.355
LEF	0.777	0.712, 6.647	.173
TNF-αi	−0.610	0.107, 2.749	.461
IL-6i	0.916	0.223, 27.966	.457
RF	0.000	0.998, 1.002	.732
Anti-CCP	−0.003	0.994, 1.000	.049
ESR	−0.005	0.977, 1.013	.595
CRP	−0.012	0.970, 1.006	.182
DAS-28 (ESR)	0.163	0.812, 1.706	.389

B: OR odds ratios, 95% CI: 95% confidence interval, *P* value: comparison between RA patients with and without PN group.

anti-CCP = anti-cyclic peptide containing citrulline, CRP = C-reactive protein, DAS28-ESR = the 28-joint disease activity score-erythrocyte sedimentation rate, ESR = erythrocyte sedimentation rate, IL-6i = interleukin-6 inhibitor, LEF = leflunomide, MTX = methotrexate, OR = odds ratio, RF = rheumatoid factor, PN = peripheral neuropathy, RA = rheumatoid arthritis, TNF-αi = tumor necrosis factor-α inhibitor.

## 7. Discussion

Rheumatoid arthritis (RA) is an autoimmune disease that affects multiple bodily systems, primarily characterized by arthritis and bone degradation. PN is a disorder of the nervous system presenting a wide range of clinical symptoms, including sensations like pain, tingling, paresthesia, and dysesthesia, as well as numbness. Clinically, PN can be classified into 2 main types: large fiber peripheral neuropathy, characterized by loss of sensation, poor balance, and weakness, and small fiber neuropathy, characterized by pain, tingling, burning sensations, and sensitivity to cold. PN is a common extra-articular manifestation of RA, with studies indicating that it can affect up to 85% of RA patients, among whom 20% display clinical symptoms related to peripheral neuropathy.^[7]^ Despite its prevalence, the exact mechanism underlying the development of PN in RA remains unclear. Some theories suggest that inflammatory cytokines may play a role in damaging peripheral nerves, while others propose vasculitis as a primary cause.^[8]^ Research indicates that patients with RA and PN exhibit reduced levels of miR-9-5p and miR-342-3p in their serum, while miR-181a levels are significantly elevated. When stimulated by inflammatory cytokines such as IL-6 and TNF-α, miR-9-5p levels decrease, leading to inhibition of its activity and subsequent apoptosis of Schwann cells. However, the hypothesis implicating inflammatory cytokines in peripheral nerve damage requires further investigation.

The present study aimed to investigate the risk factors and clinical characteristics associated with PN in RA patients, yielding several significant findings. Firstly, no significant differences were observed in gender ratio, disease duration, laboratory markers, or disease assessment scores between RA patients with PN and those without PN. This suggests that these factors may not directly contribute to PN development in RA patients. However, age was found to be significantly associated with PN, with PN patients being older compared to those without PN. This association was particularly pronounced in patients with MN involvement, indicating that age plays a crucial role in PN occurrence, especially in MN patients. These findings corroborate previous studies suggesting that age-related changes and degenerative processes may contribute to PN development in RA.^[9]^ Interestingly, some studies have reported no difference in age between patients with and without PN.^[10,11]^ The course of a disease can be influenced by various factors, including disease duration, disease activity, and treatment strategies. Additionally, our study has certain limitations. While our findings suggest potential distinctions between MN and SN groups, these comparisons must be interpreted with caution due to the limited sample size, particularly in detecting subtle associations or differences between these subgroups. Future research directions should prioritize multicenter studies with larger cohorts to validate the observed trends in MN/SN groups, coupled with long-term follow-up to monitor dynamic progression patterns of PN in RA patients. Until such validation is achieved, these subgroup analyses remain exploratory and hypothesis-generating. Despite discrepancies in the literature, our study provides valuable insights into understanding the association between peripheral neuropathies and age in RA. By fostering an open and informative discussion, we aim to further comprehend this complex relationship and its implications for clinical practice.

Furthermore, the study identified an inverse association between levels of anti-CCP antibodies and the risk of PN in RA, where lower anti-CCP titers were associated with increased PN susceptibility, particularly in patients with SN involvement. While this finding is consistent with conclusions drawn by other researchers,^[10]^ the relationship remains correlative and does not imply causality. Further mechanistic studies are warranted to elucidate whether anti-CCP antibodies actively modulate immune responses or merely reflect underlying pathophysiological processes in PN development.

The analysis of clinical symptomatology in RA patients with PN revealed that pain and numbness were the most prevalent symptoms reported by PN patients, followed by fatigue and burning sensations. Electromyography results demonstrated that the majority of PN patients exhibited sensory and motor impairments, with a higher prevalence of carpal tunnel syndrome. These findings are in line with previous studies and offer valuable insights into the clinical presentation of PN in RA,^[10]^ aiding clinicians in better understanding associated symptoms and guiding appropriate management strategies for these patients. This study focused on objective electrophysiological and serological markers in RA patients with PN. Consequently, functional impact assessments (e.g., quality of life scales, activities of daily living) were not systematically collected, which represents an important direction for future research. Future studies should incorporate patient-reported outcomes to evaluate real-world functional burden in RA patients with PN.

In summary, this study offers important insights into the factors that increase the risk of developing PN in patients with RA. Age and levels of anti-CCP antibodies emerged as key predictors for PN development. These results emphasize the significance of taking these factors into account when evaluating and treating RA patients, especially in relation to the start and advancement of PN. Further investigation is needed to clarify the precise mechanisms behind these connections and to create specific interventions for PN in RA patients, aiming to improve patient outcomes and quality of life.

## Author contributions

**Data curation:** SiHui He, Sha-Sha Hu.

**Formal analysis:** SiHui He, Jingjing Xie, Jianyong Zhang, Ertao Jia, Hongling Geng.

**Project administration:** Jianyong Zhang, Ertao Jia.

**Writing – original draft:** SiHui He, Sha-Sha Hu, Ertao Jia.

## References

[1] Gravallese EM Firestein GS. Rheumatoid arthritis - common origins, divergent mechanisms. N Engl J Med. 2023;388:529–42. 36780677 10.1056/NEJMra2103726

[2] Biswas M Chatterjee A Ghosh SK Dasgupta S Ghosh K Ganguly PK. Prevalence, types, clinical associations, and determinants of peripheral neuropathy in rheumatoid patients. Annal Ind Acad Neurol. 2011;14:194–7. 10.4103/0972-2327.85893 PMC3200043 22028533

[3] Durel CA Feurer E Pialat JB Berthoux E Chapurlat RD Confavreux CB. Etanercept may induce neurosarcoidosis in a patient treated for rheumatoid arthritis. BMC Neurol. 2013;13:212. 24373564 10.1186/1471-2377-13-212 PMC3878785

[4] Elrefaey A Memon AB. Painful small fiber neuropathy associated with teriflunomide: a case series and literature review related to teriflunomide and leflunomide. Cureus. 2023;15:e45079. 37705563 10.7759/cureus.45079 PMC10496022

[5] Tektonidou MG Serelis J Skopouli FN. Peripheral neuropathy in two patients with rheumatoid arthritis receiving infliximab treatment. Clin Rheumatol. 2007;26:258–60. 16683176 10.1007/s10067-006-0317-z

[6] Sugiura F Kojima T Oguchi T. A case of peripheral neuropathy and skin ulcer in a patient with rheumatoid arthritis after a single infusion of tocilizumab. Mod Rheumatol. 2009;19:199–203. 18987779 10.1007/s10165-008-0132-2

[7] Bayrak AO Durmus D Durmaz Y Demir I Canturk F Onar MK. Electrophysiological assessment of polyneuropathic involvement in rheumatoid arthritis: relationships among demographic, clinical and laboratory findings. Neurol Res. 2010;32:711–4. 20307377 10.1179/016164109X12581096870195

[8] Kishore S Maher L Majithia V. Rheumatoid vasculitis: a diminishing yet devastating menace. Curr Rheumatol Rep. 2017;19:39. 28631066 10.1007/s11926-017-0667-3

[9] Kaeley N Ahmad S Pathania M Kakkar R. Prevalence and patterns of peripheral neuropathy in patients of rheumatoid arthritis. J Fam Med Primary Care. 2019;8:22–6. 10.4103/jfmpc.jfmpc_260_18 PMC6396610 30911476

[10] Li Y Jiang L Zhang Z. Clinical characteristics of rheumatoid arthritis patients with peripheral neuropathy and potential related risk factors. Clin Rheumatol. 2019;38:2099–107. 30911944 10.1007/s10067-019-04521-5

[11] de Araújo Pereira F de Almeida Lourenço M de Assis MR. Evaluation of peripheral neuropathy in lower limbs of patients with rheumatoid arthritis and its relation to fall risk. Adv Rheumatol. 2022;62:9. 35317839 10.1186/s42358-022-00238-3 PMC8938971

